# Thromboelastographic monitoring of fondaparinux in surgical patients

**DOI:** 10.1186/cc12294

**Published:** 2013-03-19

**Authors:** B Martinez, R Giacomello, R Paniccia

**Affiliations:** 1 University Hospital of Udine, Italy; 2 University of Florence, Italy

## Introduction

Fondaparinux is currently the first therapeutic choice in several clinical settings such as deep venous thrombosis (DVT) prophylaxis in orthopedics or in cases of acute coronary syndromes. The main drawback of FOND is that routine monitoring is not currently available. This could be a problem during the management of critical and surgical patients, especially in cases of old patients and renal failure. The aim of this study is to evaluate the ability of thromboelastography (TEG) to determine the level of anticoagulation due to FOND in a surgical population.

## Methods

We prospectively analyzed all patients to whom elective major orthopedic surgery was consecutively performed in a 2-month period. All the patients received FOND 2.5 mg in the postoperative period according to ACCP 2012 Guidelines. Native and heparinase (hep) TEG (Haemoscope Corporation, Niles, IL, USA) tests activated with kaolin were performed using whole blood citrated samples at four times: T0, before FOND administration; T1, 2 hours after administration; T2, 17 hours after administration (half-life); T3, 24 hours after administration. The following native and hep TEG parameters were analyzed: reaction time (R), α angle, maximum amplitude (MA) and coagulation index (CI). These parameters were compared with levels of anti-Xa. Unvariate analysis and Spearman's test were applied to our data.

## Results

Eighteen patients were analyzed. Ten patients met the inclusion criteria. The mean R value increased from T1 to T3. The mean R parameter was in the normal range at any phase of the study and there was no significant differences between the R mean value at the different phases. The lowest value of R was at T1, which coincides with plasmatic peak concentration of FOND. This value did not correlate with anti-Xa mean value at T1, which showed the highest value at that time. There was significant difference between the mean native and hep R value only at T2 (*P <*0.05), native and hep α angle at T3, MA and MA hep at T2 (*P <*0.01) and CI and CI hep at T3 (*P <*0.02). Only the parameter MA had significant variation over time (*P <*0.02).

## Conclusion

R represents the time necessary to thrombin formation and in the presence of FOND we hypothesized a prolonged R time. In our population, TEG performed with citrated kaolin-activated whole blood was not able to detect prophylatic doses of FOND in every phase. On the contrary, levels of anti-Xa were able to reveal the exact pharmacokinetics of the drug. Further studies including a large number of patients are necessary.
